# The anxiolytic effects of a Valerian extract is based on Valerenic acid

**DOI:** 10.1186/1472-6882-14-267

**Published:** 2014-07-28

**Authors:** Axel Becker, Falko Felgentreff, Helmut Schröder, Beat Meier, Axel Brattström

**Affiliations:** Inst. Pharmacology and Toxicology, Otto-von-Guericke University, Magdeburg, Germany; Inst. Biotechnology, Zürich University of Applied Sciences, Wädenswil, Switzerland; Alexander Puschkin Str. 50, 39108 Magdeburg, Germany

**Keywords:** Valerian extract, Valerenic acid, Elevated plus maze, Anxiety, Binding

## Abstract

**Background:**

Valerian is commonly used for the treatment of insomnia and anxiety. Valerian extracts allosterically modulate GABA-A receptors and induced an anxiolytic activity. This activity is closely related to valerenic acid. In the present experiments it was investigated whether acetoxy valerenic acid may interfere with the anxiolytic action of valerenic acid.

**Methods:**

Situational anxiety was measured using male CD-1 mice in the elevated plus maze test after oral administration of the test substances. In addition the body core temperature was measured. For the ^3^H-GABA binding assay dissected tissue from frontal cortex of male RjHan:WI rats were used. Statistical evaluation was performed by means of the non-parametric Kruskal-Wallies H-test, followed by the two-tailed Mann–Whitney U-test.

**Results:**

Adding of acetoxy valerenic acid abolished the anxiolytic action of valerenic acid. There was no effect on body core temperature. Moreover, the valerian extract did not show any affinity to benzodiazepine binding sites.

**Conclusion:**

The determining compound for the observed anxiolytic effect of the valerian extract is its content of valerenic acid.

## Background

Valerian is commonly used for the treatment of insomnia and anxiety. *In-vitro* experiments demonstrated that valerian extracts allosterically modulate GABA-A receptors [1–3], an action related to valerenic acid [4, 5]. These results were confirmed with *in-vivo* rodent studies [1, 2].

Derivatives of valerenic acid (VA), i.e. acetoxy valerenic acid (AVA) or hydroxy valerenic acid (HVA), do not allosterically modulate GABA-A receptors, but they bind to identical binding sites [1, 4]. Valerian extract (VE) was fractionated and the fractions were tested with respect to their ability to allosterically modulate GABA-A receptors. The fractions with high VA and low AVA demonstrated the most pronounced allosteric action [5].

Recently experiments were reported in which two VEs were compared with respect to their anxiolytic activity using the elevated plus maze (EPM) test. The extracts differed in their ratio between VA and AVA. Only the extract VE-1 with the high VA and low AVA content (12:1) demonstrated a clear anxiolytic effect whilst VE-2 (AV:AVA = 1:1.5) failed in this respect [6]. From these results the suggestion comes that AVA may inhibit the anxiolytic action of VA. To further characterise the possible VA-AVA interaction, the active valerian extract VE-1 was tested again in the EPM and than AVA was added in expectation that the anxiolytic activity measured in the EPM may abolish. The applied ratio of VA to AVA corresponds to that of commercially available VEs [7]. Again, the total amount of VAs was kept constant.

Moreover, in vitro experiments have shown that derivates of VA interact with GABA receptors. Animal studies reported that central or systemic administration of GABA and GABA agonists produced hypothermia [8–10]. Therefore, the effect of VA-AVA combination on body core temperature was additionally measured.

The allosteric activity of VEs and VA was reported to occur at a binding site which is different from the benzodiazepine binding site [1, 2]. This would explain, at least in part, anxiolytic effects of VE. Therefore, for the extracts used the GABA and benzodiazepine receptor binding were determined.

## Methods

### Extract preparation

For the extract preparation (VE -1) valerian roots and rhizomes from a specifically selected species were used. A sample specimen from the selected species (HAL 115562) is preserved in the Herbarium of the Institute of Biology, Martin Luther University Halle, Germany. The dried starting material was mixed with hydro-ethanol (70% V/V) in a ratio of 1:5 at 45°C for 3½ days and then separated by filtration under 2 bar pressure. The obtained tinctures were concentrated using a rotary evaporator. The chromatogram of the obtained extract is given elsewhere [6].

### Animals

For the behavioural experiments male CD-1 mice (Charles River, Sulzfeld, Germany) were kept under controlled laboratory conditions with a light/dark cycle of 12:12 (lights on at 06.00 a.m.), temperature 20 ± 2°C, and air humidity between 55 and 60%. The animals had free access to commercial pellets (ssniff R/M-H, ssniff Spezialdiäten GmbH, Soest, Germany) and tap water. The animals were housed in groups of 10 in Macrolon III cages. After arrival, the animals were given a period of 2 weeks for habituation. At the beginning of the experiments the mice were 8 weeks old. The number of animals per group was between 12 and 18.

For the binding experiment male RjHan:WI rats (Janvier, Le Genest-Saint-Isle, France) were used. The rats were kept under controlled laboratory conditions as described above. The animals were housed in groups of 5 in Macrolon IV cages.

The work reported here was conducted in accordance with EC regulations and the National Act on the Use of Experimental Animals (Germany). The protocol was approved by the Saxony-Anhalt Committee on Animal Care (42502-2-1169).

### Elevated plus maze (EPM)

Situational anxiety was measured in the elevated plus maze test [11]. The maze was made of black polyvinyl chloride and had two open and two closed arms (50 × 10 × 40 cm) mounted 50 cm above the floor. The floor of the arms was smooth. Light intensity was 30 lux. A mouse was placed on the central platform of the apparatus facing a closed arm. A camera on the ceiling of the test room was used to score and tape the animals’ behaviours from an adjacent room for a period of 7 min. The number of entries into open and closed arms, time spent in open arms and time spent in closed arms were measured and %time in open arms (related total time 420 s) was calculated. An entry is defined as placing both forepaws into the given compartment of the maze. Anxiolytic drugs prolong the time spent on the open arms. The maze was cleaned and dried after each trial.

In a first step, using the elevated plus test maze, to further verify the hypothesis that the ratio between VA:AVA is important for the anxiolytic activity of VE, the aqueous extract VE-1 with the high VA and low AVA content (12:1) was used to replicate the anxiolytic activity of 0.5 mg/ kg [6]. Then AVA (HWI Analytik GmbH, Rülzheim, Germany) was added to the extract to change the ratios of VA:AVA from 12:1(VE-1), to 1:0.5, 1:1, and 1:1.5, respectively. The extract amount was adapted in such a way that the sum of VAs, i.e. VA plus AVA, was always identical. The different extract amounts with respect to their VA:AVA ratios are given in Table 1. VEs (0.5/kg, with respect to the total sum of VA and AVA) were administered orally (application volume 0.1 ml/10 g body weight) using a mouse gavage feeding needle (24 gauge, FST, Heidelberg, Germany). As control a 0.9% saline solution was administered. A positive control has already been reported [6]. Following the EPM, body core temperature (BCT) was measured with a digital thermometer (ama digit) manufactured by Amarell GmbH (Kreuzwertheim, Germany).

**Table 1:** **VA and AVA in different VE compositions and the calculated amount for oral administration**

	VA:AVA ratio	VA mg/ml	AVA mg/ml	VA + AVA mg/ml	VE amount to be administered 0.5 mg VA + AVA
VE - 1	12:1	1.165	0.0983	1.2633	0.400 ml
VE	1:0.5	1.165	0.583	1.748	0.286 ml
VE	1:1	1.165	1.165	2.33	0.215 ml
VE	1:1.5	1.165	1.75	2.915	0.172 ml

For that purpose the lubricated probe (Ø 1 mm) was gently inserted 3 cm into the rectum.

All behavioural tests were performed in the light period between 8.00 a.m. and 2.00 p.m. The animals were randomly assigned for testing.

### Binding assay

^3^H-GABA (SA – 1,43 TBq/mmol) and ^3^H-flunitrazepam (SA – 1,85 TBq/mmol) were obtained from PerkinElmer (Boston, USA), diazepam and chlordiazepoxide from AWD.pharma GmbH (Dresden, Germany). The tested VEs were identical to those extracts VE-1 and VE-2 used in the earlier study [6]. **Assay:** For the ^3^H-GABA binding assay tissue (dissected rat frontal cortex) was homogenized in 50 mM Tris–HCl buffer pH 7.4 containing 120 mM NaCl, 5 mM KCl, 2 mM CaCl_2_ and 1 mM MgCl_2_; for ^**3**^**H**- flunitrazepam it was homogenized in 50 mM Tris–HCl buffer pH 7.4 containing 1 mM EGTA, 10 mM EDTA and 3 mM MgCl_2_, centrifuged (50.000 g, 15 min) and washed twice with the corresponding buffer. The resulting pellet was resuspended with buffer. Aliquots of the crude membrane suspension (50 μg protein) were incubated for 45 min at 37°C with 2.5 nM ^3^H-GABA or 1 nM ^3^H-flunitrazepam at 0–4°C for 60 min. Specific binding was calculated by subtracting non-specific binding – defined as that seen in the presence of 1 μM unlabelled GABA or 1 μM diazepam or chlordiazepoxide – from the total binding obtained with radioligand alone. In parallel VE-1, VE-2 and AVA were used for all binding assays. The incubation was terminated by addition of ice-cold buffer and rapid filtration through glass fibre filters. The filters were washed, dried, a scintillation cocktail was added, and radioactivity was counted using a β-counter (Beckman Coulter, Germany). The EC_50_ values were determined by the addition of unlabelled drugs (GABA, chlordiazepoxide, diazepam, AVA, VE-1 and VE-2) over a wide concentration range (10^-10^ to 10^-4^ M).

### Statistics

Due to non-Gaussian distribution of the data obtained in the elevated plus maze, for statistical evaluation the non-parametric Kruskal-Wallies H-test, followed by the two-tailed Mann–Whitney U-test were used. Body core temperature was analyzed using ANOVA. The threshold for significance was set at p < 0.05.

## Results

### Elevated plus maze

**Figure 1:**
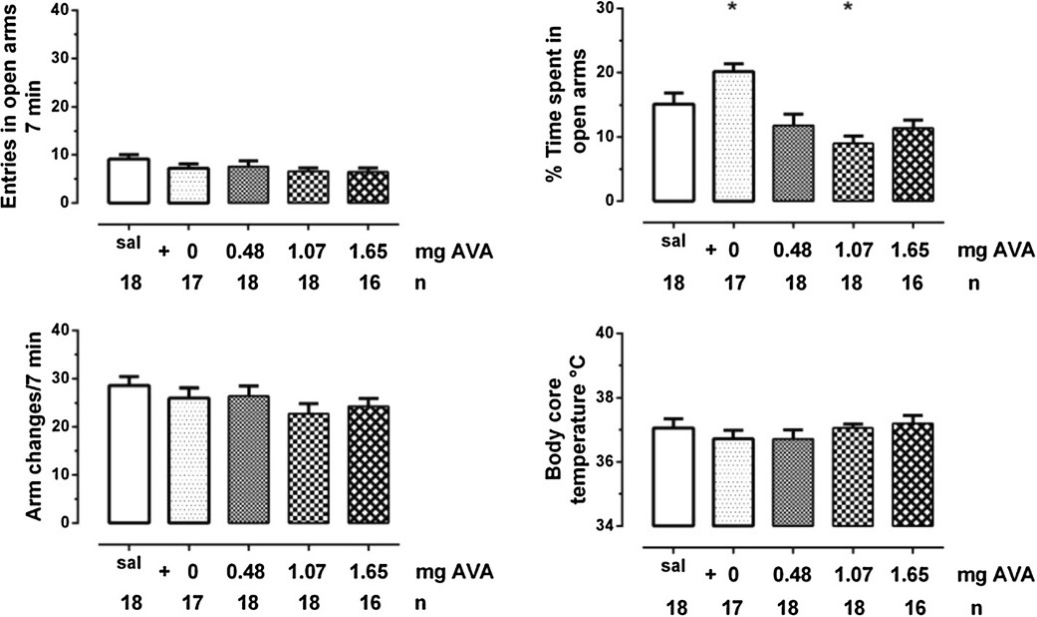
**% Time spent in the open arm, total arm changes, number of entries in open arms (median ± deviation of the median) and body core temperature (mean ± sem) for the different 0.5 mg/kg valerian extract – acetoxy valerianic combinations (AVA).** The amount of added AVA to VE is indicated (mg AVA), n equals the number of animals used. *p < 0.05 in comparison to saline.

For the animal groups treated with 0.5 mg VAs /kg with added AVA a significant effect was found (Χ^2^_4, 87_ = 24.37, p < 0.001). The control animals (treated with saline) spent 15.15 ± 1.76% time in the open arm. Animals which had received VE (total amount of VAs 0.5 mg/kg; VA:AVA = 12:1) exhibited extended times in the open arm (20.16 ± 1.24%; U _2, 18, 17_ = 89.0, p = 0.035). This is a significant anxiolytic effect (Figure 1). The results of the other animal groups, which had received VE with different ratios of VAs (VA : AVA = 1:0.5 or 1:1.5) were either in the range of the saline control group or demonstrated just the opposite effect, i.e. an anxiogenic action (VA:AVA = 1:1; U _2, 18, 18_ = 79.5, p = 0.008).

The number of total arm changes (Χ^2^_4, 87_ = 4.89, p = 0.298, Figure 1) and entries in open arms (Χ^2^_4, 87_ = 7.39, p = 0.12, Figure 1) were similar in all experimental groups.

### Body core temperature

The BCT remained for all experimental groups in the control range (0.5 mg/kg, F _4, 82_ = 0.725, p = 0.725). In one animal BCT was reduced to be below 34°C (0.5 mg/kg = 1). The result of this animal was disregarded for data evaluation.

### Binding assay

Both of the tested valerian extracts did not bind at ^3^H-flunitrazepam labelled binding sites of the benzodiazepine receptors, while diazepam (EC50: 0.40 ± 0.05 μM) as well as chlordiazepoxide did (EC50: 0.64 ± 0.08 μM). At the crude synaptosomal membranes GABA (EC50: 1.82 ± 0.21 μM) as well as VE-1 (EC50: 5.61 ± 1.68 μM) and VE-2 (EC50: 10.04 ± 1.34 μM) compete with ^3^H-GABA at the GABA binding site, whilst AVA did not. The binding affinity was more expressed with VE-1 compared to VE-2.

## Discussion

Oral administration of a valerian extract (VA: 0.5 mg/kg) with high VA and very low AVA content (12:1) produced a significant anxiolytic activity. As shown in Figure 1 the experimental groups did not significantly differ in the two parameters reflecting locomotor activity, i.e. entries in open arms and total arm changes. This indicates that the anxiolytic effect is not affected by locomotor activity. This result is in accordance with a recent report in which diazepam (1 mg) was included as positive control [6]. Adding AVA to this valerian extract abolished the anxiolytic action, which underlines the competition of both acids at the binding sites [1, 4]. The ratios of VA and AVA in the range of 1:0.5, 1:1 and 1:1.5 are the most frequent ratios reported for standard preparations of valerian root [7], whereas hydroxy-valerenic acid as an AVA metabolite is not important in this context [12]. Interestingly, the dose–response curve for the anxiolytic effect of the different VA: AVA combinations are not linear. An elevated concentration of AVA was reported to close the open GABA-A channel and thus limits its action [4]. This might explain the dose–response curve in regard to the parameter %time spent in open arms as shown in Figure 1. However, the behavioural effect of increasing AVA concentrations has to be considered in relation to the concentration of its VA competitor.

Whether or not the anxiolytic action of VE is restricted to its VA acid content is an open question, since valerenol, 6-methylapigenin and linarin, i.e. other components of the extract, also demonstrate activity at the GABA channel [1, 13, 14]. These results however were obtained from *in-vitro* experiments and confirmation from *in-vivo* studies is still lacking.

Moreover, VE was more active than the detected amount of VA [1, 15], a fact which may point to the participation of other components or indicates that other components of the extract influence VA bioavailability, since bioavailability of VA after oral administration was 33.7% [16].

From other *in-vitro* experiments a VE mediated inhibition of cerebral GABA degradation enzymes was reported, whereby the VE activity may have been additionally enhanced [17]. Moreover, VE and VA also interact with glutamate receptors, from which an anxiolytic effect can also be induced [18]. For both of the latter reports confirmation from *in-vivo* conditions is not as yet available.

Although the combination with different amounts of AVA did reduce the anxiolytic effect of VE-1 (Figure 1), we did find no effect of VE-1 or the combinations with AVA on BCT. It was found that intraperitoneal injection of diazepam (1.5 - 6 mg/kg) decreased the BCT of the rats [9]. This implies that the hypothermic effect is basing on doses of this substance which are supramaximal in relation to its anxiolytic effects. Considering the dose-effect relation concerning the anxiolytic effect found reported in a recent study [6] this might explain that lack of alterations in BCT underlining the therapeutical usefulness of valerian extract.

Short-acting benzodiazepines are the first line option for the treatment of anxiety disorders. Besides their limitations regarding treatment duration and adverse events, a correlation to being a risk factor for dementia was reported quite recently [19]. Benzodiazepines bind to the α + γ- subunits at the GABA-A channel [20]. The tested valerian extracts (VE-1, VE-2) failed in this respect. However, there are other subunits for binding [21] and other ways of GABA receptor modulation. It may be worthwhile to mention in this context that VA binds to the β + α- subunit [1, 4], i.e. its action occurs not at the benzodiazepine binding site and may therefore basically provide clinically relevant advantages particularly regarding impairments in the domain of cognition. The interaction between VA and AVA in the EPM can not be explained based on the binding data presented here. For this reason more elaborate tests directly at the β + α- subunits are necessary.

## Conclusion

In conclusion, the VA content of VE seems to be an important factor for the observed anxiolytic activity. AVA may modulate this action. Hitherto based on the monograph for valerian the sum of VA and AVA contents is expressed as VA (Ph Eur. monograph 0453). Considering the presented results, this declaration is misleading. VA and AVA should be separately declared to provide a basis for the expected anxiolytic activity of that VE. The observed anxiolytic VE activity is not mediated via the benzodiazepine binding site. VA as a natural compound may belong to a new class of substances with the capability to allosterically modulate the GABA-A channel.

## Abbreviations

AVA: Acetoxy valerenic acid
BCT: Body core temperature
EPM: Elevated plus maze
HVA: Hydroxy valerenic acid
SA: Specific activity
VA: Valerenic acid
VE: Valerian extract.
